# Electromagnetic Encoders Screen-Printed on Rubber Belts for Absolute Measurement of Position and Velocity

**DOI:** 10.3390/s22052044

**Published:** 2022-03-05

**Authors:** Ferran Paredes, Cristian Herrojo, Ana Moya, Miguel Berenguel Alonso, David Gonzalez, Pep Bruguera, Claudia Delgado Simao, Ferran Martín

**Affiliations:** 1 CIMITEC, Departament d’Enginyeria Electrònica, Universitat Autònoma de Barcelona, 08193 Bellaterra, Spain; cristian.herrojo@uab.es (C.H.); ferran.martin@uab.es (F.M.); 2 Eurecat, Centre Tecnològic de Catalunya, Parc Científic i de la Innovació TecnoCampus, 08302 Mataró, Spain; ana.moya@eurecat.org (A.M.); miguel.berenguel@eurecat.org (M.B.A.); claudia.delgado@eurecat.org (C.D.S.); 3 Hohner Automation, P.I. Cal Batlle, 17400 Breda, Spain; dgonzalez@encoderhohner.com (D.G.); bruguera@encoderhohner.com (P.B.)

**Keywords:** electromagnetic encoders, screen-printing, motion control, microstrip technology

## Abstract

This paper presents, for the first time, an absolute linear electromagnetic encoder consisting of a rubber belt with two chains of screen-printed metallic inclusions (rectangular patches). The position, velocity, and direction of the belt (the moving part) is determined by detecting the inclusions when they cross the stator (the static part). The stator is a microstrip line loaded with three complementary split ring resonators (CSRRs), resonant elements exhibiting a resonance frequency perturbed by the presence of inclusions on top of them (contactless). The line is fed by three harmonic signals tuned to the resonance frequencies of the CSRRs. Such signals are generated by a voltage-controlled oscillator (VCO) managed by a microcontroller. The sensed data are retrieved from the pulses contained in the envelope functions of the respective amplitude modulated (AM) signals (caused by the belt motion) generated at the output port of the line. One of the signals provides the absolute belt position, determined by one of the chains, the encoded one. The information relative to the velocity and motion direction is contained in the other AM signals generated by the motion of the other chain, periodic, and thereby, uncoded. The spatial resolution of the system, a figure of merit, is 4 mm. Special emphasis is devoted to the printing process of the belt inclusions.

## 1. Introduction

Motion control is today present in a wide variety of industrial scenarios, such as robotics, the automotive and aeronautics industry, the textile industry, logistics, the food industry, mechatronics, space, and medical instrumentation, to cite some examples. In any motion control system, a sensor is used to provide the necessary feedback signal to the controller and actuator (e.g., a step motor), in order to eventually compensate for any potential motion error. Proximity, displacement (either linear or angular), velocity, and acceleration are typical motion variables that need to be retrieved in many applications. For that purpose, there are a wide variety of motion sensors on the market. In particular, optical encoders (either linear or angular) are used in many industrial systems (e.g., servomechanisms, conveyor belts, pointing mechanisms, reaction wheels, elevators, etc.) [1,2,3].

Optical encoders, although very accurate, exhibit an intrinsic drawback, i.e., a limited robustness against the effects of dirtiness, grease, and pollution (present in most industrial systems). By contrast, electromagnetic (or microwave) encoders, reported recently [4,5,6,7,8,9,10], are tolerant against such pernicious effects, since these devices utilize microwaves for sensing. Magnetic encoders, proposed in [11,12], are also robust against hostile environments, but such sensors are relatively complex, since they need inductive coils. In the electromagnetic encoder systems reported so far, the movable element (a dielectric disk in rotary encoders and a dielectric tape in linear encoders) should be attached to the moving target, e.g., a shaft (rotary encoders) or a conveyor belt (linear encoders). In this paper, we investigate the possibility of using the moving target as the encoder, provided it is made of a dielectric material, in particular, rubber. This is of interest, since many industrial and logistic systems utilize belts made of rubber (e.g., conveyor belts, elevator belts, etc.). Thus, the encoder presented in this paper is implemented by screen-printing (using commercial conductive inks) chains of rectangular patch inclusions on a rubber belt, specifically a commercial elevator belt.

Nevertheless, besides the implementation of the encoder in rubber material by means of conductive ink using screen-printing (for the first time), there are other aspects that represent an innovation with regard to the previously proposed encoders. Probably, the most important one concerns the fact that in the synchronous system proposed in this paper, where the encoder velocity, quasi-absolute position, and motion direction can be determined, the three harmonic signals needed for that purpose are generated by means of a VCO managed by a microcontroller. Moreover, the microcontroller processes and separates the three signals providing that information. In the encoders presented in [8,9,10], a synchronous reading is also proposed, but the proof-of-concept demonstrators are based on obtaining the required (envelope function) signals by injecting the necessary harmonic signals sequentially, so that each signal is inferred at different times, rather than from the processing action of the microcontroller. On the other hand, the encoder presented in [7] exhibits very good resolution, thanks to the use of transversely oriented metallic strips, but it does not operate synchronously. However, in the paper [10], the system works similarly to [7], but it is able to provide the direction of motion and operates synchronously (nevertheless, the resolution is not as good as in [7]). For that purpose, the reader used three double stub pairs, thereby representing a significant size for the reader. Concerning the encoder system presented in [8], it was the precursor of the one proposed in this paper, but we have demonstrated for the first time system its functionality by including the necessary elements for retrieving the signals containing the relevant information, i.e., the VCO, the microcontroller, and the associated circuitry. Moreover, in [8], the encoder was implemented in a rigid microwave substrate by means of a drilling machine. Let us finally mention, in regard to other encoders available in the literature, that those reported in [4,5] are rotary, rather than linear, and are not absolute encoders but incremental-type encoders. Nevertheless, in the prototype presented in [5], the direction of motion (clockwise or counterclockwise) can be detected.

There are many other systems for the measurement of displacements, velocities, and proximity. For example, there are sensors based on coupling modulation [13,14,15,16,17] and frequency variation [18], where a resonant (movable) element is displaced over a transmission line based structure (typically, the resonator in motion is displaced in a plane parallel to the one of the lines, but there are also realizations, where the resonator displaces vertically [17,19]). However, these systems offer a very limited input dynamic range, as compared to linear electromagnetic encoders, where such dynamic range is potentially unlimited. On the other hand, there many displacement and proximity sensors based on the Hall Effect [20,21,22,23,24,25], and there are commercially available devices [26]. In general, these devices offer excellent resolution, even by considering significant input dynamic ranges, but they are based on the use of magnets. The system proposed in this paper cannot compete against optical encoders and Hall effect sensors in terms of resolution, but, in many applications, the resolutions demonstrated in this paper suffice, in particular in industrial scenarios (elevators, conveyor systems, etc.).

## 2. Sensor System

**Figure 1:**
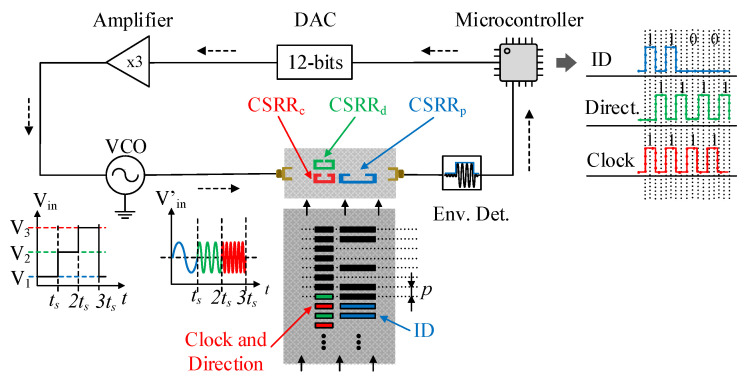
Sketch of the encoder sensor system.

The proposed linear displacement and velocity sensor consists of two parts: the movable belt with two screen-printed chains of rectangular metallic inclusions (the encoder) and a planar microwave structure (stator) able to detect the inclusions when the belt is in relative motion with regard to the stator. The sketch of the complete system is depicted in Figure 1. One of the encoder chains is periodic and is used to determine the velocity (velocity chain). The other chain is encoded, that is, certain inclusions are present at the same axial positions of the inclusions of the velocity chain (‘1’ logic state), whereas other inclusions are not present (‘0’ state). With this chain (position chain), it is possible to determine the absolute position of the encoder by “reading” the state (bit) corresponding to that position. That bit, plus the bits corresponding to the previous N − 1 positions, provide a unique code that identifies univocally the position of the encoder. In order to guarantee that any subset of N bits does not repeat, it is necessary to encode the whole position chain following the De Bruijn sequence [27]. The variable *N* is the number of bits necessary to univocally determine the different positions of the encoder (in turn determined by the length of the encoder, *L*, and by the spatial resolution, *p*, or chain period), i.e., *L/p* < 2*^N^*. Note also that the instants of time necessary to read the position chain are determined by the velocity chain (also called clock chain).

**Figure 2:**
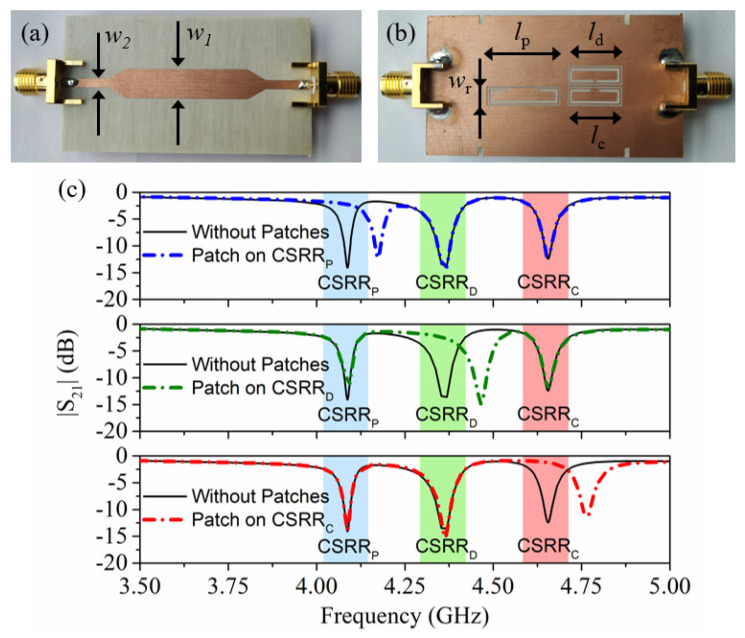
Photograph of the stator. (**a**) Top and (**b**) bottom. (**c**) The frequency response of the stator when the patches rely on the top of each resonator. The stator was fabricated on a *Rogers RO4003C* substrate with a thickness of *h* = 0.81 mm, dielectric constant *ε_r_* = 3.38, and loss factor tan δ = 0.0027. The dimensions are: transmission line widths *w*_1_ = 6.4 mm and *w*_2_ = 1.9 mm; CSRR widths *w_r_* = 2.9 mm (for the three resonators); resonators lengths *l_c_* = *l_d_* = 10.5 mm and *l_p_* = 14.5 mm; ring splits *s_d_* = 0.4 mm, *s_c_* = 1.6 mm and *s_p_* = 6.2 mm; and CSRR slot width *c* = 0.5 mm. The sub-index *c*, *d*, and *p* in the variables corresponding to the resonator’s length and ring splits are used to differentiate the CSRRs.

The static part of the system is a microstrip transmission line loaded with three CSRRs tuned to different frequencies (see Figure 2). In order to determine the belt velocity, it is necessary to inject a harmonic signal into the input port of the line tuned to the resonance frequency of the resonator identified as CSRR_c_. This frequency, *f*_0,*c*_, varies when a patch of the velocity chain is contactless on top of the CSRR_c_. The presence of the patch modifies the transmission coefficient at *f*_0,*c*_ (see Figure 2c). Consequently, by encoder motion, an amplitude modulated (AM) signal is generated at the output port of the line, and the instantaneous velocity of the belt is determined from the time lapse between adjacent peaks in the envelope function of that AM signal. The resonator designated as CSRR_p_ is devoted to determining the ID code of the position chain, and for that purpose, a harmonic signal tuned to *f*_0,*p*_, the resonance frequency of the unloaded CSRR_p_, is required. Finally, the resonator called CSRR_d_, with resonance frequency *f_0_,_d_*, is also sensitive to the velocity chain, but it is located in such a way that the generated envelope function is either lag or lead with regard to the envelope function of the AM signal with carrier frequency *f*_0,*c*_. Thus, it is possible to determine the motion direction.

**Figure 3:**
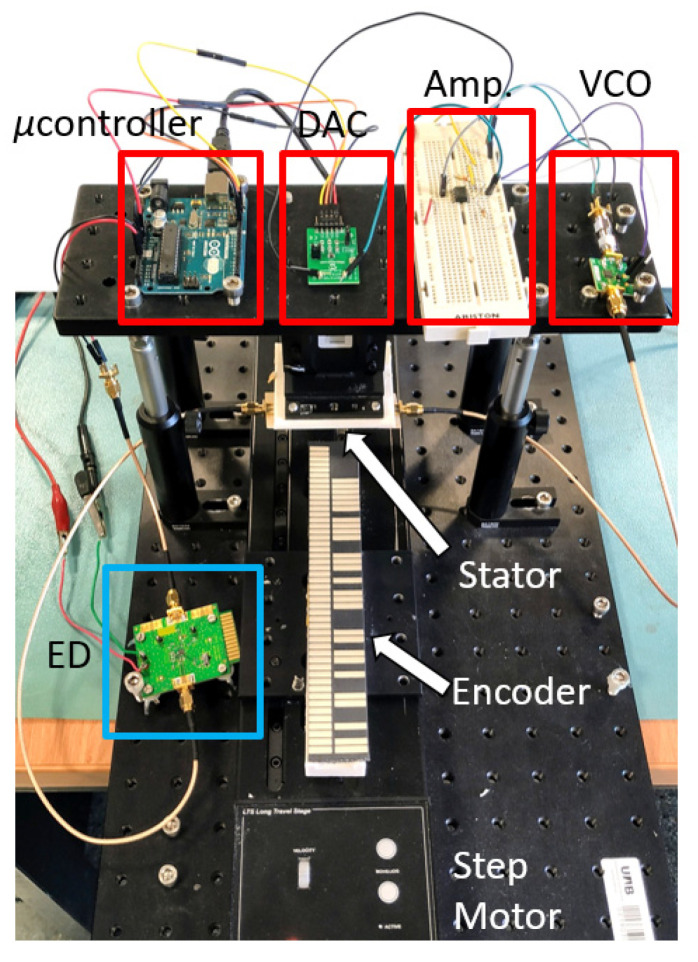
Photograph of the experimental setup.

For the generation of the harmonic signal, the *HMC391LP4* VCO (Analog Devices, Norwood, MA, USA) was used. The control voltage of this component is managed by the *ATmega328* microcontroller (Atmel Corporation, San Jose, CA, USA) (*Arduino* development platform). As shown in Figure 1, the microcontroller output was connected to the 12-bit *MCP4725* DAC (Sparkfun Electronics, Boulder, CO, USA), followed by an amplifier stage, in order to cover the full operation range of the VCO. A linear displacement system (model *Thorlabs LTS300/M* (Thorlabs Inc., Newton, NJ, USA)) was employed to displace the encoder over the stator. The air gap, the separation between the stator and encoder, was set to 1 mm. Finally, the *ADL5511* envelope detector was connected between the output port of the stator and the input port of the microcontroller. The photograph of the experimental setup is depicted in Figure 3.

The procedure to generate the signals and process the data was as follows. First, the microcontroller set a specific control voltage *V*_1_ (so that the VCO generated a tone at *f*_0,*p*_) and read the output signal of the envelope (AM) detector. After the sample period, *t_s_* = 1.25 ms, the control voltage was changed to *V*_2_ (corresponding to the unmodulated tone *f*_0,*d*_), and the procedure was repeated (the microcontroller retrieved the signal given by the AM detector) until the end of the sample period, 2*t_s_*. Finally, at 2*t_s_*, the voltage was set to *V*_3_ (with the VCO giving the tone at *f*_0,c_), and this voltage was kept until *t* = 3*t_s_*. This sequence was constantly repeated while the encoder was in motion, and the three AM modulated signals (envelope functions) were obtained. Note that the total sweep time was 3*t_s_* = 3.75 ms. Let us assume that it is reasonable that the time period between the crossing of two adjacent patches above the resonators was at least 10 times higher than 3*t_s_* = 3.75 ms (this is a conservative value taking into account the sampling Nyquist criterion). This gives 37.5 ms, and, taking into account that the distance between adjacent patches (period) in the considered encoder was 4 mm, the maximum encoder velocity was *v_max_* = 4 mm/37.5 ms = 106 mm/s (note that in our case, the considered encoder velocity was set to 40 mm/s, as mentioned later). However, it should be mentioned that depending on the microcontroller, the time *t_s_* can be reduced to few μs. Thus, in this case, the velocities of the encoder can be, at least, two orders of magnitude higher.

It should be mentioned that the frequencies of the harmonic signals necessary for system functionality were chosen in the vicinity of 4 GHz as a tradeoff. That is, reducing the frequency increases the size of the resonators of the static part of the sensor, or reader (i.e., the size of the CSRRs) and, consequently, the size of the patches, with a penalty in terms of encoder resolution. Thus, for sensor resolution, operation at high frequencies is preferred, but, in this case, all the associated sensor electronics were more complex and expensive.

## 3. Encoder Design and Fabrication

The encoders were implemented by screen-printing the two chains of patch inclusions on a rubber belt. The considered belt had a steel core in order to provide mechanical strength against possible tensile stress. Nevertheless, the presence of the steel core did not seem to have an appreciable effect on sensor functionality, particularly in the envelope detector (as will be shown in Section 4). It is believed that the rubber material present between the steel core and the patches sufficed to minimize the effects of the steel core. The length, width, and thickness of the belt were 20 cm, 3 cm, and 3 mm, respectively. The metallic patches were designed, along with the stator, considering the maximum printable width of the belt (3 cm), and taking into account that two chains must be assembled.

**Figure 4:**
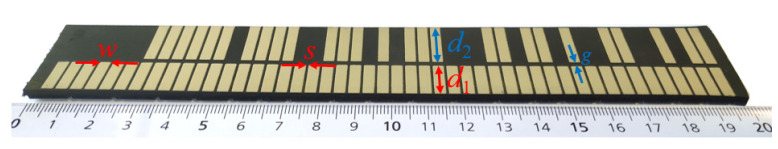
Photograph of the 48-bits encoder with clock chain. The dimensions are: *d*_1_ = 11.5 mm; *d*_2_ = 15.9 mm, *w* = 3 mm, *s* = 1 mm, and *g* = 1.9 mm. The chain period is *p* = 4 mm. Note that *L*/*p* = 48; thus, *N* = 6.

The screen-printing process consisted of the deposition of pastes or inks printed on a substrate through a screen. The screen, based on polyester threads, was placed at a certain distance from the substrate, and it contained the layout to be printed. When a scraper was pressed down, the screen reached the substrate, depositing the ink. A screen-printing technique must consider three factors: (i) the layout to be printed in terms of the resolution of strips and slots, (ii) the desired conductivity of the ink when printed, and (iii) the substrate. In the present design, the resolution of the rectangular inclusions was not critical. However, it was important to determine the adhesion of the ink to the substrate, in this case the rubber belt, as well as the conductivity of the silver ink. Thus, previous to implementing the inclusions, some patterns consisting of strips of different widths and length were considered, in order to measure the conductivity and to verify the adhesion of the ink. The patterns were screen-printed using the Norcote ELG conductive silver ink. Then the ink was UV cured by a halogen light (5 s at 500 W), and the printed belt was finally introduced in an air flow oven (10 min at 130 °C) to cause thermal curing. The ink was correctly adhered to the rubber belt; therefore, the conductivity was measured (*σ* ≈ 1 × 10^6^ S/m), and this value ensured the system functionality. Finally, the metallic inclusions were screen-printed using the same process, and the fabricated encoder was depicted in Figure 4.

## 4. Results

**Figure 5:**
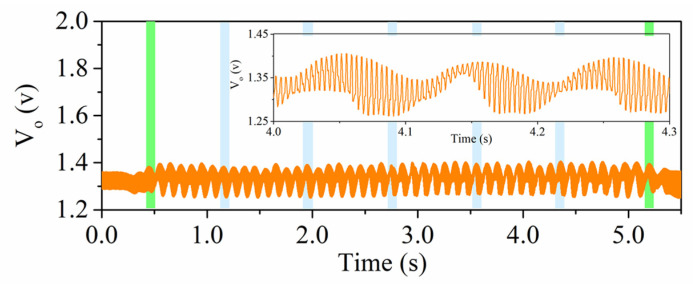
Measured signal at the output of the envelope (AM) detector.

**Figure 6:**
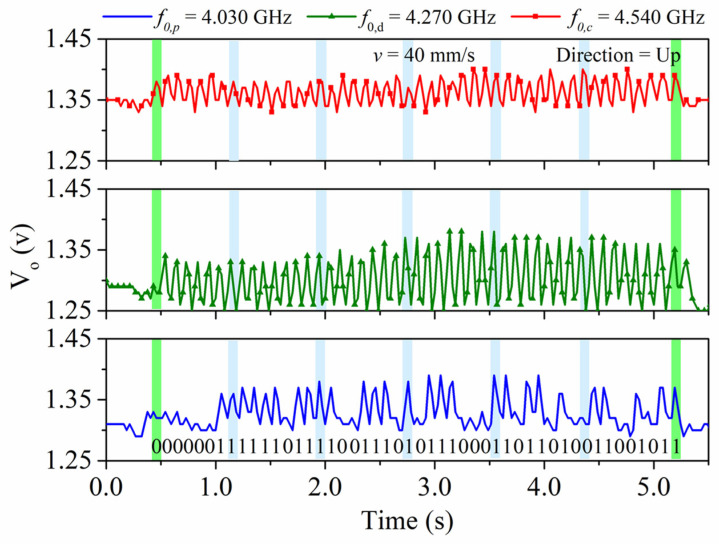
Measured envelope function for the clock, direction, and position signals after the microcontroller separated the signal of Figure 5.

The functionality of the proposed encoder system was validated by means of the experimental setup of Figure 3. The encoder was displaced at a velocity of v = 40 mm/s in the upwards direction. Figure 5 shows the measured signal at the output port of the envelope (AM) detector, which contained the three AM demodulated signals (envelope functions). The microcontroller was in charge of processing and separating the signals (Figure 6). A good synchronism between the clock and the ID code was visible. The direction signal was delayed with regard to the clock signal, thus indicating that the encoder moved upwards. The separation between peaks was 100 ms, which corresponded to an encoder velocity of 40 mm/s, since the period of the metallic inclusions (resolution) was *p* = 4 mm. Finally, the peaks in the ID code signal perfectly correlated with the presence of rectangular patches in the position chain (see Figure 4). With these results, the functionality of the system was validated. Nevertheless, it can be appreciated that the discrimination capability of the systems was limited since the voltage difference between the logic states ‘0′ and ‘1′ was not very significant. One possibility to improve this aspect is to include an amplifier stage after the VCO. By doing this, the difference will increase, thereby favoring discrimination. However, this aspect was left for a future work.

Another important aspect that determined the functionality of the system was the vertical distance between the patches of the encoder and the static part (reader), particularly the CSRRs. The patches should be located at a maximum vertical distance (or air gap) that guarantees that such patches perturb the resonance frequency of the resonant elements (CSRRs) of the static part of the sensor when they are on top. In our case, the nominal distance was 1 mm, but it was concluded from electromagnetic simulations that tolerances in the air gap between 0.5 mm and 1.5 mm were compatible with system functionality. Indeed, the proposed system, despite this nominal air gap, was subjected to certain variations in the air gap distance, unavoidable in practice. However, it should be mentioned that as the air gap increased, the modulation index decreased, thereby having a negative impact on the capability of the system to discriminate between the ‘0’ and the ‘1’ logic states.

## 5. Conclusions

In conclusion, a linear displacement and velocity sensor based on an electromagnetic encoder was proposed. The system was able to provide the absolute position and the instantaneous velocity of the movable part (the encoder) with regard to the static part (stator). Such functionality was achieved by designing the encoder with two chains of metallic patches. One chain was periodic, with all the patches present at their predefined positions. The other chain was provided with a certain identification code (achieved by the presence or absence of patches at their predefined positions in the chain). This code followed the so-called De Bruijn sequence, where any subset of *N* bits did not repeat along the whole encoder, *N* being the number of bits necessary to univocally determine the 2*^N^* different positions of the encoder belt (*N* = 6, in our case). Nevertheless, the main relevant aspect of the present contribution concerned the implementation of the encoder on a belt made of rubber, by means of screen-printing, using commercial conductive inks. Despite the limited electromagnetic characteristics of the considered materials, as compared to those of previous electromagnetic encoders, typically implemented on rigid microwave substrates, or on flexible polymeric substrates, the functionality of the system was validated by considering a displacement of the encoder at constant velocity. It can be envisaged that the proposed encoders may find application in many industrial systems equipped with dynamic (movable) elements made of rubber, such as conveyor or elevator belts, among others.
